# Prebiotic competition and evolution in self-replicating polynucleotides can explain the properties of DNA/RNA in modern living systems

**DOI:** 10.1186/s12862-020-01641-4

**Published:** 2020-06-26

**Authors:** Hemachander Subramanian, Joel Brown, Robert Gatenby

**Affiliations:** 1 Cancer Biology and Evolution Program, Tampa, FL 33612 USA; 2 Integrated Mathematical Oncology Department, H. Lee Moffitt Cancer Center, 12902 Magnolia Dr, Tampa, FL 33612 USA; 3 Present Address: Department of Physics, National Institute of Technology, Durgapur, West Bengal India

**Keywords:** Prebiotic evolution, Autocatalytic self-replicators, DNA, RNA, Origins of life, Origins of natural selection

## Abstract

**Background:**

We hypothesize prebiotic evolution of self-replicating macro-molecules (Alberts, Molecular biology of the cell, 2015; Orgel, Crit Rev Biochem Mol Biol 39:99-123, 2004; Hud, Nat Commun 9:5171) favoured the constituent nucleotides and biophysical properties observed in the RNA and DNA of modern organisms. Assumed initial conditions are a shallow tide pool, containing a racemic mix of diverse nucleotide monomers (Barks et al., Chembiochem 11:1240-1243, 2010; Krishnamurthy, Nat Commun 9:5175, 2018; Hirao, Curr Opin Chem Biol 10:622-627), subject to day/night thermal fluctuations (Piccirilli et al., Nature 343:33-37, 1990). Self-replication, like Polymerase Chain Reactions, followed as higher daytime thermal energy “melted” inter-strand hydrogen bonds causing strand separation while solar UV radiation increased prebiotic nucleobase formation (Szathmary, Proc Biol Sci 245:91-99, 1991; Materese et al., Astrobiology 17:761-770, 2017; Bera et al., Astrobiology 17:771-785, 2017). Lower night energies allowed free monomers to form hydrogen bonds with their template counterparts leading to daughter strand synthesis (Hirao, Biotechniques 40:711, 2006).

**Results:**

Evolutionary selection favoured increasing strand length to maximize auto-catalytic function in RNA and polymer stability in double stranded DNA (Krishnamurthy, Chemistry 24:16708-16715, 2018; Szathmary, Nat Rev Genet 4:995-1001, 2003). However, synthesis of the full daughter strand before daytime temperatures produced strand separation, longer polymer length required increased speed of self-replication. Computer simulations demonstrate optimal polynucleotide autocatalytic speed is achieved when the constituent nucleotides possess a left-right asymmetry that decreases the hydrogen bond kinetic barrier for the free nucleotide attachment to the template on one side and increases bond barrier on the other side preventing it from releasing prior to covalent bond formation. This phenomenon is similar to asymmetric kinetics observed during polymerization of the front and the back ends of linear cytoskeletal proteins such as actin and microtubules (Orgel, Nature 343:18-20, 1990; Henry, Curr Opin Chem Biol 7:727-733, 2003; Walker et al., J Cell Biol 108:931-937, 1989; Crevenna et al., J Biol Chem 288:12102-12113, 2013). Since rotation of the nucleotide would disrupt the asymmetry, the optimal nucleotides must form two or more hydrogen bonds with their counterpart on the template strand. All nucleotides in modern RNA and DNA have these predicted properties. Our models demonstrate these constraints on the properties of constituent monomers result in biophysical properties found in modern DNA and RNA including strand directionality, anti-parallel strand orientation, homochirality, quadruplet alphabet, and complementary base pairing. Furthermore, competition between RNA and DNA auto-replicators for 3 nucleotides in common permit states coexistence and possible cooperative interactions that could be incorporated into nascent living systems.

**Conclusion:**

Our findings demonstrate the molecular properties of DNA/RNA could have emerged from Darwinian competition among macromolecular replicators that selected nucleotide monomers that maximized the speed of autocatalysis.

## Background

Living systems, uniquely in nature, acquire, store and use information to maintain a highly ordered state while remaining far from thermal equilibrium. The molecular carriers of heritable information, RNA in virtually all living systems consist of the same four nucleotides. The properties of DNA are similarly universal among cells. Thymine substitutes for uracil and deoxyribose for ribose in going from RNA to DNA [1]. Multiple investigations of plausible prebiotic chemistry suggest it likely produced a complex, racemic mixture of multiple different nucleobases [2–5] including “unnatural” [6] nucleobases that can produce replicating DNA [7, 8] or RNA [9]. Thus, there is no clear reason for U,C,G,A, and T to be the only constituent [10] monomers generated by prebiotic chemistry [11–13]. It is reasonable to assert that they emerged non-randomly from many potential alternative nucleotides. Furthermore, while polynucleotides in living systems possess physicochemical properties that permit encoding and transmitting information [14, 15], they also retain biophysical properties, such as strand directionality [16, 17], anti-parallel strand orientation, complementary base pairing and homochirality [17, 18], that confer no obvious evolutionary advantage. In fact, as outlined below, some of these properties seem to be disadvantageous to modern life.

If the constituent nucleotides of living systems do, in fact, represent one of many possible combinations, the universal properties of RNA and later DNA may simply represent the heritage (i.e. “frozen in time” [8]) of the first replicators. That is, the Last Universal Common Ancestor (LUCA) [19, 20] may have inherited rather than evolved this specific system of RNA, DNA, and proteins along with their constituent monomers. Here we propose an alternative hypothesis in which the properties of RNA and DNA and their specific nucleotide monomers were deterministically selected by prebiotic evolutionary dynamics in competing self-replicating polynucleotides. In this, we may see the first example of natural selection promoting adaptations to solve one problem (the struggle for existence between polynucleotides) and enabling unintended consequences for later developments (chemical specialization and then cellular life). Thus, the RNA and DNA present in LUCA did not occur by chance but represented a dominant macromolecular self-replicating “species” that outcompeted others and was optimally adapted to prebiotic evolutionary selection pressures.

## Results

### General model of prebiotic evolution

We assume that early earth had widespread biophysical characteristics that ^will result from^ would permit prebiotic Darwinian dynamics among self-replicating polynucleotides. Central to our hypothesis is simply the day/nigh cycle which causes an external, diurnal energy fluctuations sufficient [21, 22] to provide alternating sources and sinks of Gibbs free energy which has both enthalpic and entropic components (ΔG = ΔH -TΔS). Thus, during daylight, we assume thermal energy (or enthalpy – energy at constant pressure) was sufficient to overcome energetic barrier (i.e. the hydrogen bonds between strands), whereas at night, the energy release from stable covalent bond formation (during synthesis of a daughter strand) dissipated as heat. We note that many primordial energy sources have been proposed [22, 23] and hydrothermal vents have been recently favoured. While the energy changes during day/night cycles would likely have been smaller than those associated with thermal vents [23], we favour this scenario because diurnal cycles impose regular variations that are necessary for self-replication as well as temporal constraints that impose Darwinian selection for optimizing the dynamics of self-replication. Furthermore, the UV radiation in sunlight could catalyse formation of nucleotides bases [4] which would then be available for strand synthesis at night. Finally, diurnal fluctuations of temperature provide a relatively constant frequency while the amplitude of the temperature variations may vary because of local weather and seasonal changes. The former provides a regular informational metric on time while the latter may impose potential threats or opportunities. Thus, self-replicators could, for example, measure local conditions and, by using their “knowledge” of the current time within the diurnal cycle, “predict” and adapt to conditions during the remainder of the cycle. The role of cyclical forcing functions in promoting dynamic self-assembly and network formation has been noted in multiple physical systems [24].

### Modelling autocatalytic polymers in prebiotic conditions

Schrödinger first noted self–replicating polymers capable of information storage [25], requiring monomers that form two kinds of bonds among themselves. Stronger, thermodynamically near-irreversible bond between monomers are necessary for polymerizing monomers. Weaker, thermodynamically reversible bonds between monomers of the template and daughter strands permit autocatalytic self-replication. Furthermore, auto-catalysis requires that monomers forming new strands do so preferentially on a template compared to spontaneous synthesis. Self-replication on a template is favoured when hydrogen bonds of two contiguous monomers binding to the template catalyse the formation of covalent bonds between them.

### The requirement for symmetry breaking

The technical details of our computer simulations have been published [26]. The evolutionary dynamics outlined above will include two sets of conflicting demands. First, optimal self-replicating polymers must balance stability which favours longer polymers [27] and short replication times which favours short strands. Second, long polymers with short replication times will need to simultaneously select for both monomer acquisition *and* monomer retention. However, monomer acquisition is favoured by lowering the hydrogen bond kinetic barrier, which maximizes the probability that a monomer in solution will attach to the template. However, monomer retention is favoured by increasing the kinetic barrier which decreases the probability of monomer separation from the template strand. This is similar to the dynamical models of cytoskeletal filament polymerization [28–30], although important differences exist between these two models. The filamental molecules of actin or microtubule are structurally constrained to grow or shrink only at the ends, whereas, DNA polymerization can happen anywhere on the template strand, but is similarly constrained only through our model.

**Fig. 1:**
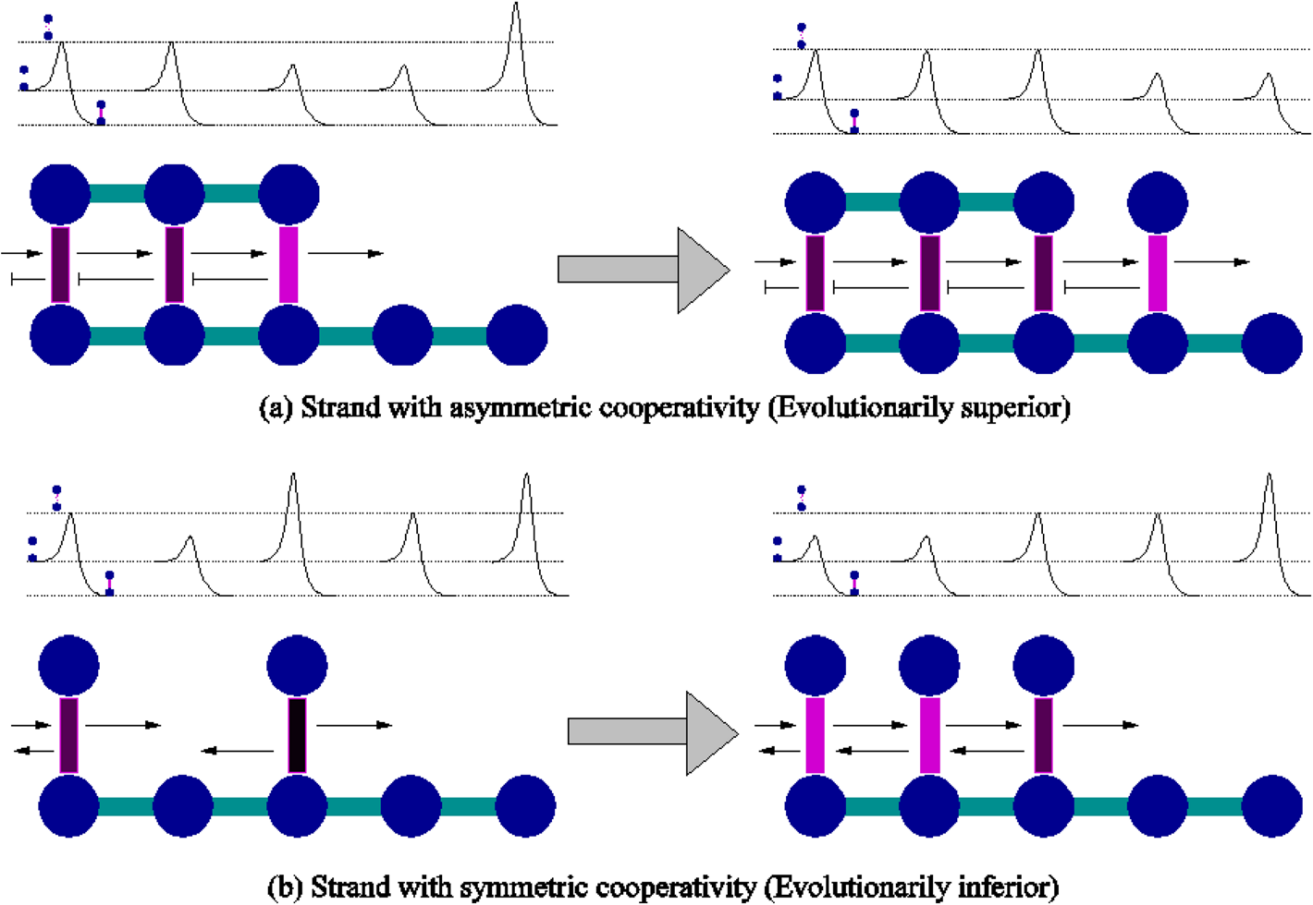
**a** The energy diagrams above the polymers show the heights of the kinetic barriers for bond formation/dissociation in various regions. Dark- vertical bars are hydrogen bonds with high kinetic barrier, while lighter bars denote bonds with lower kinetic barriers. The balls-and-sticks diagram of polymer growth illustrates the fact that asymmetric cooperativity enables faster strand growth by lowering kinetic barrier for bond formation to the right. Asymmetric kinetic influence of a hydrogen bond on adjacent monomers (raising the barrier of the left bond and lowering it for the right) optimizes strand elongation as well as increases the duration of monomer bonding to the template strand to increase the probability of covalent bond formation. **b** With symmetric kinetic influence, hydrogen bonds that are away from the growth front (second bond from left) have lower kinetic barriers. Thus, monomers are drawn away from growth front, resulting in lower growth rate. This makes symmetric replicators evolutionarily inferior (From Ref [26], with permission)

As Anderson observed [31, 32], a physical system typically responds to imposed, incompatible forces by breaking symmetries. Similarly, our computational model (Fig. 1, adapted from [26]) shows how a left-right asymmetry in monomers and their resulting polymers satisfies the two conflicting selection forces. An asymmetric monomer, upon forming a hydrogen bond with its counterpart on the template strand, asymmetrically influences the hydrogen bond kinetic barrier for adjacent nucleotides. In forming a new strand, the kinetic barrier for bond formation/dissociation is lower on one side and higher on the other [33, 34]. Decreasing the barrier for the nucleotide bonding/dissociation to the right (say) of a pre-existing bond would increase the probability of a monomer in solution binding to the template (speed of association). Similarly, increasing the barrier for the nucleotide on its left would reduce the probability of separation of an already attached monomer (stability of retention). Finally, monomer symmetry breaking also imposes strong directionality on the self-replicating polymer so that the addition of free nucleotides to the complementary strand must occur from right to left (or vice-versa) rather than haphazard and simultaneous binding at all sites which, a priori, might appear to be a faster mechanism of self-replication.

### Experimental observations of directional asymmetry in modern RNA

If the ribozyme properties of RNA evolution preceded the development of full living systems, we anticipate the “fittest” and most abundant extant RNA species would likely have been integrated into primordial life forms. This is supported by the following observations:

### Nucleotide properties

**Fig. 2:**
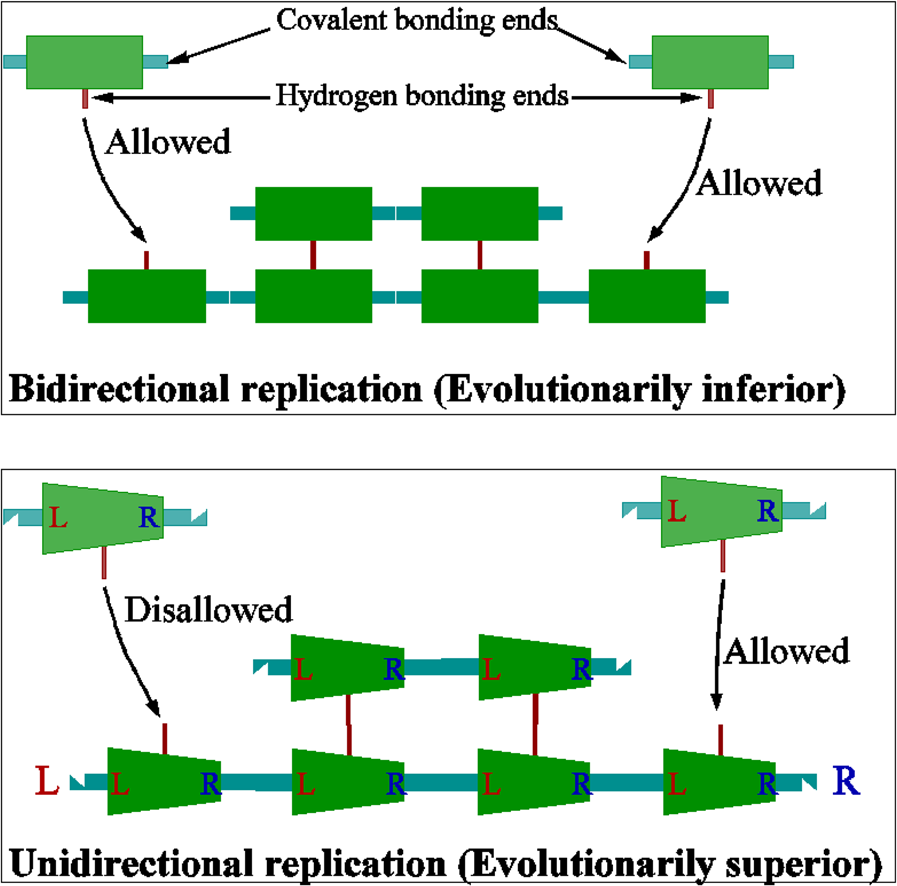
(top) An illustration of bidirectional self-replication. Symmetric kinetic influence helps in bidirectional strand construction and requires left-right symmetric monomers. (Bottom) Structural instantiation of asymmetric cooperativity requires left-right asymmetric polymers to distinguish between left and right. Asymmetric monomers instantiate asymmetric cooperativity and thus simultaneously satisfy the two conflicting requirements of fast monomer acquisition and their retention for successful self-replication. This leads to the evolutionary superiority of unidirectional self-replicators

The most obvious prediction of our hypothesis is that all nucleotides in modern RNA should show right-left directional asymmetry. It lowers the free nucleotide binding energy on one side and increases it on the other. Note that rotation of a nucleotide along its strand would reverse this effect and prevent the sequential addition of nucleotides on the growing strand. To prevent this rotational disruption, nucleotides must bind with their counterpart on the template strand with two or more hydrogen bonds (Fig. 2). In fact, all constituent nucleotides in modern RNA and DNA form 2 or 3 hydrogen bonds with their counterpart on the opposite strand.

### Strand directionality

As shown in Fig. 2, the asymmetric effects of monomers on the hydrogen bonds [35] lowers the kinetic barrier at the adjacent empty site increasing the probability that a diffusing monomer will bind. This produces a directionality of strand self-replication that is maintained in all modern organisms. Both the “reading” and “duplication” of double stranded RNA and DNA proceed only along the 3′-5′ direction of the template and not in the kinetically improbable reverse direction.

### Evolutionary dynamics in self-replicators

As noted above, self-replicating polynucleotides in a constrained environment such as a tide pool would have competed for monomers. In addition, cycling temperatures which promoted the sequence of strand separation and replication also imposed selection for replicative speed and accuracy. That is, complete autocatalysis required the new strand to fully replicate each nucleotide on the template prior to the onset of warmer daytime temperatures, which would produce strand separation. Furthermore, erroneous integration of a nucleotide without asymmetric properties would reduce the replicative speed of the daughter strand reducing the probability it will subsequently replicate removing it from the lineage. Note, however, there is no restriction on mixing nucleotides that possess the necessary asymmetric effects during replication. In total, these dynamics produce both environmental selection forces, a mechanism of inheritance that is imperfect thus introducing heritable variation into the replicating population.

In the Darwinian competition among autocatalytic replicators, mathematical models find that directional strand replication will be faster than will any other method of replication. For example, while simultaneous binding of nucleotides to all sites on the empty strand would intuitively seem to allow faster strand synthesis, the dwell time of each nucleotide on the template would be too low to allow covalent bonds to form consistently.

Curiously, double-stranded RNA today only exists in the form of viruses indicating it can store information in a manner that we propose for the first replicators. The absence of double stranded RNA in prokaryotic and eukaryotic life probably reflects subsequent evolutionary optimization in which DNA evolved to provide a more biochemically stable molecule for storing information while RNA became specialized to translate this information into specific sequences of amino acids in a polypeptide. As described below, the proposed evolutionary dynamics would include competition between autocatalytic RNA and DNA for 3 of the 5 nucleotides. This would allow scenarios of stable co-existence so that both “species” of polynucleotides would have been available to be incorporated in early living systems.

### Experimental demonstration of nucleotide asymmetry, DNA/RNA unzipping energies

A simple prediction of the proposed right-left asymmetric influence on adjacent monomer bond energy is corresponding asymmetry in bond breaking. Experimental observations [35–37] demonstrated that the average force required to unzip double stranded RNA and DNA is significantly greater from one end than the other, consistent with the expected asymmetry.

### Evolution of anti-parallel strand orientation and heteromolecular base-pairing

**Fig. 3:**
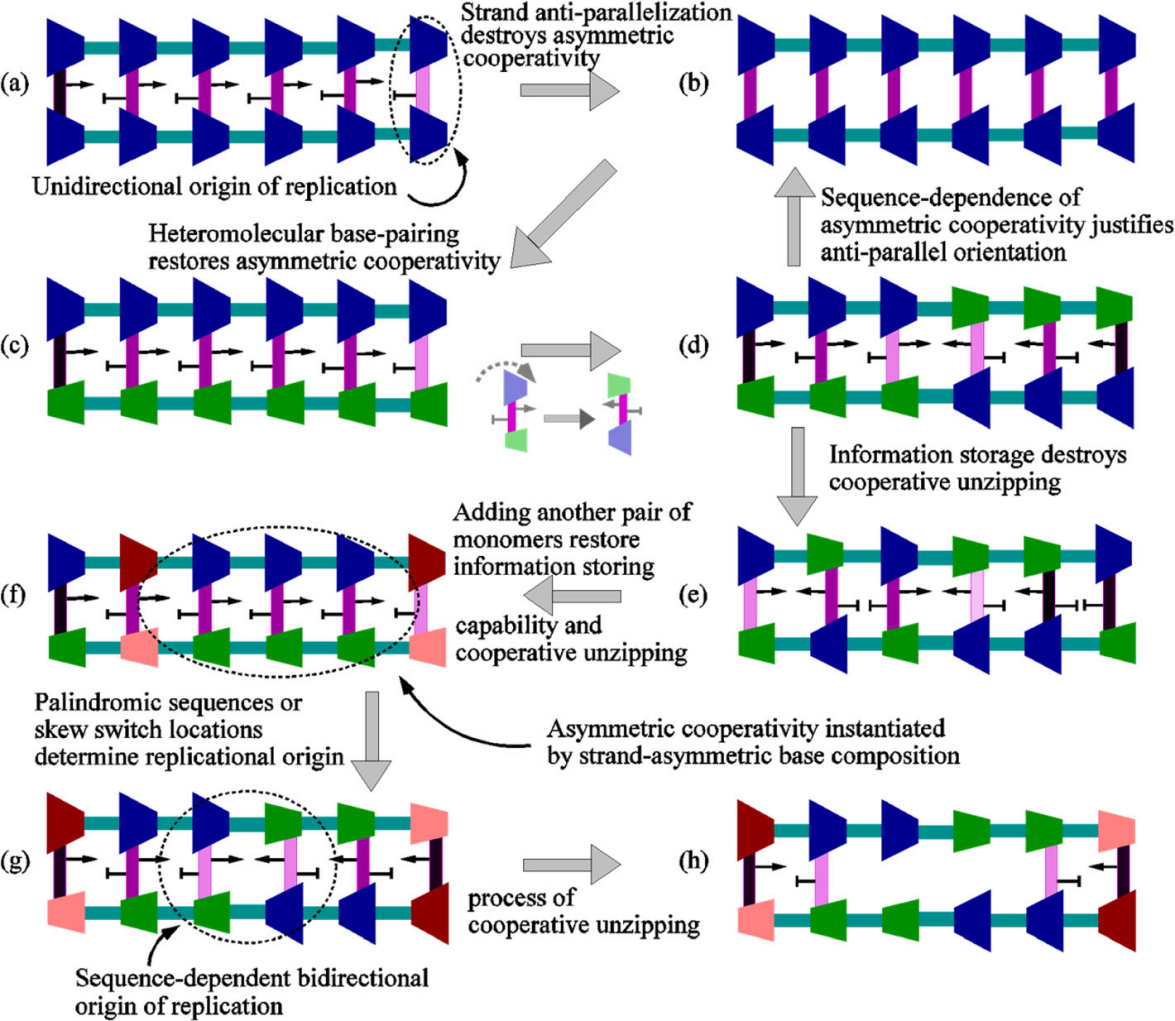
**a** Parallel strand orientation of the duplex DNA freezes the mode of asymmetric cooperativity along the entire length of the strand, reducing the kinetic barrier of the hydrogen bond in the right adjacent monomer and decreasing that of the left adjacent monomer. Both the strands of the duplex DNA act in concert to incorporate asymmetric cooperativity. **b** Anti-parallel orientation with homo-molecular base-pairing destroys the asymmetric cooperativity because the two identical strands oppose each other’s asymmetric cooperativity mode due to their opposing orientations. **c** Reinstating asymmetric cooperativity requires the breaking the symmetry of homo-molecular base-pairing. Due to differences in the strengths of asymmetric cooperativity from the two strands made of different kinds of monomers, a complete cancellation of asymmetric cooperativity is avoided, leaving a resultant, comparatively weaker sequence-dependent asymmetric cooperativity. **d** Thus, heteromolecular base-pairing and anti-parallel strand orientation allows for sequence-dependent asymmetric cooperativity mode, leading to simultaneous replication of multiple disjoint segments independently, increasing the rate of replication. Simultaneous replication is possible because multiple segments can be unzipped independently, due to their different asymmetric cooperativity modes. **e** With just two monomers, information storage and unzipping kinetics are coupled, resulting in the former adversely affecting the latter. **f** Introduction of another pair of monomers decouples the two. Storing information does not adversely affect unzipping kinetics (**g**). **h** Low kinetic barriers in the middle of the double strand allows for rapid unzipping of the double strand during replication initiation, thus serving as origin of replication

As strand length increases, the time for unidirectional replication will similarly increase thus imposing additional selection pressures. We propose, as with prior adaptive strategies, autocatalytic nucleotides “solved” the trade-off of polymer length and replication time through symmetry breaking. As shown in Fig. 3, anti-parallel strand orientation, together with heteromolecular base-pairing results in sequence-dependence of asymmetric effects on adjacent hydrogen bond kinetic barriers allowing replication to occur simultaneously at multiple locations (see [33] for full details). The first three base-pairs of Fig. 3 (d) are right-asymmetrically cooperative, whereas the next three base-pairs are left-asymmetrically cooperative. This allows these two portions of the strand to replicate simultaneously and independently of each other, thus increasing the rate of replication. The alternative option would be an RNA/DNA parallel strand duplex that has no local switching of the modes of asymmetric cooperativity. Such parallel-stranded DNA have been demonstrated to form under physiological conditions. Such a replicator would be evolutionarily inferior because the parallel-strand RNA would have to unzip in a single, continuous, and sequential order. Replication of such a polymer would take far longer and the acquisition of monomers would be correspondingly slower. Such a replicator, while possible, would be outcompeted by those with different modes of asymmetric cooperativity.

### Quadruplet alphabet

The unzipping of strands of a duplex heteropolymer will be faster if strands are populated predominantly by one type of nucleotide as shown in Fig. 3. Any scrambling of this arrangement will adversely affect unzipping kinetics. Thus, fast unzipping selects for single strands composed of the same monomer. Clearly, the information content of DNA/RNA composed of a single nucleotide is insufficient for translation to proteins. It is possible that mixing of nucleotides occurred only after replicators evolved the next level of complexity involving chemical specialization between polypeptides and polynucleotides for catalysis and information storage, respectively. We hypothesize that evolution solved this issue by introducing another pair of nucleotides. This allowed RNA/DNA strands to simultaneously set the mode of asymmetric cooperativity and store information in a single strand.

Here we speculate that the primary selection force is thermal energy needed for melting of the double strand in a changing environment. The regular diurnal cycles importantly allowed a predictable temporal variation in temperatures that likely represented an initial information source for the replicators. However, while the frequency of the cycle remained relatively constant, the amplitude would change frequently due to weather conditions and seasonal effects. It seems reasonable to expect changes in the minimum and maximum temperatures during cycles would alter the separation and replication kinetics of DNA and RNA.

In considering the possible benefit of polymers formed of different nucleotides, it is notable that the energy needed to separate Adenine and Uracil (and later Thymine) with two hydrogen bonds is less than that for Cytosine and Guanine with 3 hydrogen bonds. This leads to the hypothesis that mixtures of these two types of nucleotides permitted heritable phenotypic plasticity that allowed the polynucleotides to undergo autocatalysis despite seasonal and regional variations in day and night temperatures. Furthermore, these variations allow replicators with a quadruplet alphabet to adapt to depth-dependent variations in temperature fluctuations in pools of water and perhaps variations in pH and concentrations of ions and minerals within the tidal pools. Note that this primitive information storage could lead to other phenotypic properties of single strands determined by the *sequence* of monomers such as 3-dimensional folding to perform enzymatic function in response to environmental changes.

While this hypothesis is speculative, it does make experimentally testable predictions about the evolutionary roles of multiple nucleotides in self-replicators.

### Homochirality

Homochirality is one of the most intriguing properties of RNA/DNA molecules. We propose [38] chirality in self-replicators emerged from the requirement that monomers bind to the growing daughter strand in a specific orientation to maintain unidirectionality. Fast daughter strand synthesis requires directional monomers to arrive in a specific orientation on the template for hydrogen bonding. Thus, achiral monomers would be capable of bonding in multiple possible orientations with respect to the template strand, some of which will not allow for unidirectional strand elongation. Elimination of such symmetries would favour one invariant configuration of a chiral monomer. Hence, the strand could be D only or L only, but not a combination.

### Co-evolution of DNA and RNA replicators

The proposed evolutionary dynamics allows formation of both DNA and RNA replicators. As noted above, each “species” will have different biophysical properties. Nevertheless, they will need to compete for 3 of the 5 constituent nucleotides while being subjected to identical environmental conditions and selection forces. We note that, under some scenarios, these pre-biotic evolutionary dynamics would permit equilibrium states in which both species co-existed. Furthermore, under some conditions of changing environmental conditions, they may have co-evolved mutually beneficial interactions that promoted cooperative dynamics that optimized survival for both species. These could have served as the precursors for the information dynamics of DNA and RNA in fully living systems. We anticipate this will be a subject of future investigations.

## Discussion

There is extensive interest in the dynamics that led to the development of living systems on earth. There is a general consensus that RNA provided heritable information for early living systems [1]. Here we espouse the RNA-first world. It is also clear that the interactions of free nucleotides with the template strand is critical in selecting the properties of the nucleotides that persist in modern living systems [39]. Here we investigate evolutionary dynamics that could have led to these strand replication dynamics and subsequent selection for the autocatalytic polynucleotides that ultimately became available as mechanisms of inheritance in living system.

Here we begin by assuming that prebiotic chemistry produced mixtures of monomers leading to random polymerization. However, only 5 are present in modern organisms. Here we propose that UCGA and later TCGA were integrated by legacy into early living systems due to prebiotic evolutionary dynamics among self-replicating polymers that initially formed stochastically. Thus, many of the properties of RNA/DNA are not novel adaptations meant to optimally serve cell-based life, but rather properties that served as adaptations for the first replicators. Those with these properties outcompeted all others and became the de facto ancestors of us. Subsequent living systems had to use and build upon the properties of DNA and RNA made available to them by prebiotic evolution.

We propose a plausible spatially constrained environment such as a tide pool that is subject to cyclical fluctuations in thermal energy due to the day/night temperature variations. Similar to Polymerase Chain Reactions, this allows strand separation in warm temperatures and replication in cooler conditions. In these conditions, self-replicators will compete for nucleotides. They would be simultaneously subjected to selection for longer strand length and shorter replication time. We propose, as with any physical system subject to opposing forces, this selects for symmetry breaking. Specifically, it selects for monomers that asymmetrically alter the hydrogen bond kinetic barrier for adjacent nucleotide bonding with their partner on the template strand – properties found in all 5 nucleotides in modern living systems. Interestingly, the substitution of uracil in RNA for thymidine in DNA suggests that they were subject to slightly different selection forces – possibly because flexibility for autocatalysis was a critical selection factor for RNA while polymer stability was more critical for DNA [40, 41]. These dynamics produced dominant RNA and DNA self- replicator species composed only of the 5 nucleotides that had the property of “asymmetric cooperativity.” Thus, as fully living systems developed, they did not use random polynucleotides but rather those made available to them from selection that occurred during pre-biotic evolution.

We demonstrate, similar to prior studies [39], that the templating between strands leads to hetero-molecular base-pairing, anti-parallel strand orientation, quadruplet alphabet and homochirality, all of which are observed in the DNA of modern organisms. Finally, we note that these dynamics suggest that the “origin” of evolution by natural selection preceded the origin of fully living systems.

## Conclusion

We conclude that the properties found in the DNA and RNA of all living organisms are not the result of chance inclusion in the Last Universal Common Ancestor. Rather, the specific nucleotides and replicative dynamics of the DNA/RNA available to early cells arose through Darwinian competition of polynucleotide self-replicators in environmental conditions likely present in the primordial earth. Thus, the DNA and RNA in modern living systems via evolutionary dynamics that occurred *prior to* the presence of fully living systems.

## Methods

### Aim

Examine evolution of self-replicating DNA and RNA molecules under conditions likely present in primordial earth.

### Setting (initial conditions)

Our proposed evolutionary dynamics occur in tide pools, likely common in primordial earth. Tide pools provide weakly dispersive, spatially constrained liquid environment containing multiple monomer species generated by prebiotic chemistry [40, 42–44]. We assume the nucleotides were synthesized within the tidal pool via reactions initially at equilibrium. Thus, as products of these reactions were consumed in formation of polynucleotides, more would be synthesized. Thus, to the extent that the reaction pathways “competed” for substrate, those that generated nucleotide most favourable for polymer formation would become the primary consumers.

We then subject the pool to regular, diurnal cycles near the melting temperatures of polynucleotides. The average Archean ocean temperature is estimated to be 26-35 °C but may have reached 70 °C in some locations [41, 45] and likely varied over space and time. Other initial conditions such as the reducing atmosphere, the pH, salinity, and ion concentrations in the primordial oceans [27] may have contributed to the dynamics of prebiotic chemistry and polymer autocatalysis.

Within these conditions, the diurnal thermal cycle applied regular and predictable perturbations to the environment. The critical role of cyclical thermodynamic fluctuations (as opposed to stochastic fluctuations or a thermodynamically constant state) in developing information and converting energy to order (decreased entropy) has been widely recognized [46].

We hypothesize higher daytime temperatures, similar to Polymerase Chain Reactions (PCR), provided sufficient thermal energy to melt hydrogen bonds causing the separation of polymer strands. Simultaneously, daytime UV-irradiation from sunlight could promote nucleobase formation [4]. As thermal energy decreased during night hours, each single strand could replicate as the free monomers formed hydrogen bonds with counterparts on the template strand, leading to covalent bonds between adjacent monomers and the synthesis of a daughter strand [14].

Evolution by natural selection requires heritable phenotypic variation and environmental constraints that limit proliferation such that the replicative success of an organism is governed by its properties and those of competing organisms. We propose these conditions are met as self-replicating RNA or its ancestral macromolecules, within the physical constraint of a tide pool, compete for available monomers. In addition, we note the diurnal cycle also imposes constraints on replicative speed. That is, successful completion of each daughter strand synthesis must occur prior to onset of daytime temperatures and strand separation. Thus, these prebiotic evolutionary dynamics would impose selection for fidelity and speed of replication, efficiency of substrate utilization, and stability (persistence) under local environmental conditions.

Finally, fluctuating water levels in tidal pools permitted influx of new monomers and efflux of replicators. This latter resulted in dispersal of replicators so that successful variants could colonize new pools that lack self-replicating species or were populated by replicators with inferior properties. This weak coupling of subpopulations could accelerate and promote natural selection similar to Wright’s shifting balance theory [47].

### Mathematical models and computer simulations

The mathematical models and details of computer simulations are discussed in detail in references [26]. All code used in the simulations is available from the link provided below.

## Data Availability

The MATLAB code used in the simulations is available from https://github.com/HemachanderTBio/MarkovDNA The link also includes a README file that explains what the code does and how to use it.

## Abbreviations

DNA: Deoxyribonucleic acid
RNA: Ribonucleic acid
T: Thymine
G: Guanine
C: Cytosine
A: Adenine
U: Uracil
LUCA: Last Universal common ancestor
PCR: Polymerase chain reaction
